# Emerging roles of protein palmitoylation and its modifying enzymes in cancer cell signal transduction and cancer therapy

**DOI:** 10.7150/ijbs.72244

**Published:** 2022-05-09

**Authors:** Zhuang Liu, Mingming Xiao, Yaqi Mo, Han Wang, Yamei Han, Xu Zhao, Xue Yang, Zhenxing Liu, Bo Xu

**Affiliations:** 1 Department of Biochemistry and Molecular Biology, Key Laboratory of Breast Cancer Prevention and Therapy, Ministry of Education, Tianjin Medical University Cancer Institute and Hospital, National Clinical Research Center for Cancer, Key Laboratory of Cancer Prevention and Therapy, Tianjin, Tianjin's Clinical Research Center for Cancer, Tianjin 300060, China; 2 Chongqing Key Laboratory of Intelligent Oncology for Breast Cancer, Chongqing University Cancer Hospital and Chongqing University School of Medicine, Chongqing 400030, China

**Keywords:** Palmitoylation, Palmitoylase, Depalmitoylase, Signal transduction, DNA damage response

## Abstract

Protein palmitoylation is an increasingly investigated form of post-translational lipid modification that affects protein localization, accumulation, secretion and function. Recently, emerging findings have revealed that protein palmitoylation is crucial for many tumor-related signaling pathways, such as EGFR, RAS, PD-1/PD-L1 signaling, affecting the occurrence, progression and therapeutic response of tumors. Protein palmitoylation and its modifying enzymes, including palmitoylases and depalmitoylases, are expected to be new targets for effective tumor treatment. Recognizing the significance of palmitoylation modification on protein stability, localization and downstream signal regulation, this review focuses on the regulatory roles of protein palmitoylation and its modifying enzymes in tumor cell signal transduction, aiming to bring new ideas for effective cancer prevention and treatment.

## 1. Introduction

**Figure 1:**
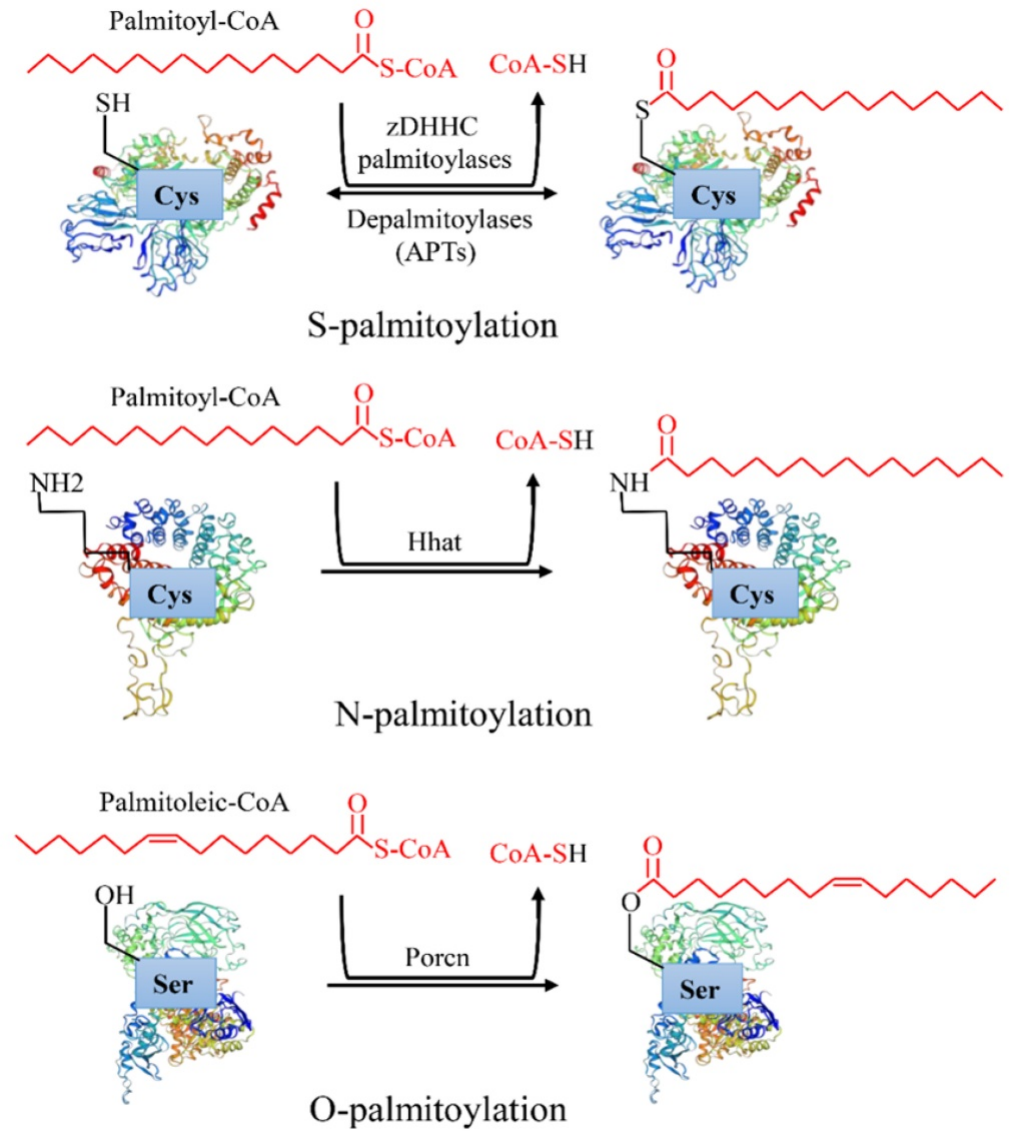
Types of protein palmitoylation: *S*-palmitoylation, *N*-palmitoylation and *O*-palmitoylation.

Protein post-translational modifications refer to the chemical modification of a given protein after translation, which endows it with a variety of physiological functions 1. Palmitoylation is an essential lipid modification, which regulates protein localization, accumulation, secretion, and function by altering protein affinity to the membrane 2. Palmitoylation was first reported in 1979 3. After more than 40 years of exploration, increasing evidence has revealed that palmitoylation is linked to a variety of human diseases, especially cancers 4. There are three types of palmitoylation: *S*-palmitoylation, *N*-palmitoylation and *O*-palmitoylation (Figure 1). Among them, *S*-palmitoylation is the main modification type 4. According to statistics, *S*-palmitoylation can occur in more than 4,000 proteins 3. *S*-palmitoylation refers to the 16 carbon fatty acid palmitate covalently binds to the specific cysteine (Cys) residues side chain of a protein through the labile thioester bond 5. Under certain conditions, the thioester bond hydrolyzes, the palmitate disassociates the Cys, and the protein is depalmitoylated 6. *S*-palmitoylation is a reversible process in which proteins can cycle between palmitoylation and depalmitoylation forms in a time range of seconds to hours 7. *N*-palmitoylation occurs when a fatty acid palmitate is linked to Cys at the N-terminal of a protein by a stable amide bond 4, while *O*-palmitoylation refers to a palmitoylation process in which the monounsaturated form of palmitate (cis Δ9 palmitate) binds to the hydroxyl group of serine or threonine by an oxyester linkage. In comparison with *S*-palmitoylation, *N*-palmitoylation and *O*-palmitoylation are relatively rare and irreversible 8. They occur mainly in secreted proteins when entering the endoplasmic reticulum, such as hedgehog 9 and Wnt 8 proteins.

**Figure 2:**
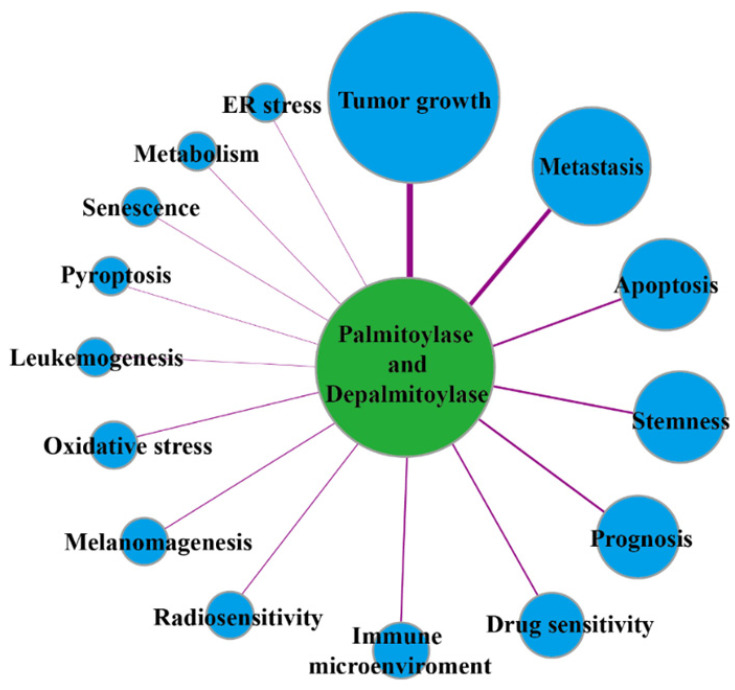
The links of palmitoylases and depalmitoylases with cancer.

Protein palmitoylation is regulated by palmitoylase and depalmitoylase, which are closely associated with tumor initiation, growth, development, therapeutic efficacy and patient prognosis (Figure 2). At present, known palmitoylases mainly include members of the zinc finger DHHC-type-containing (zDHHC) palmitoylase family, Porcupine (Porcn), hedgehog acyltransferase (Hhat) and other related enzymes 10. zDHHC family proteins are named because they all contain the conserved DHHC(Asp-His-His-Cys) enzyme activity domain. In mammals, zDHHCs consist 23 members: zDHHC1-24 (skipping zDHHC10), which catalyze the protein *S*-palmitoylation reaction 11, 12. Except for zDHHC5, zDHHC20 and zDHHC21, which are mainly located in the plasma membrane, most zDHHC palmitoylases are located in the Golgi apparatus and endoplasmic reticulum, which are the main sites for palmitoylation of proteins in mammalian cells 13. Porcn is a *O*-acyltransferase family protein that catalyses the *O*-palmitoylation of 19 Wnt proteins, and it plays a crucial role in regulation of the Wnt protein interaction with its receptors, Wnt protein secretion, and signal transduction 14. Hhat is also a membrane-bound *O*-acyltransferase family protein, but it mainly catalyzes *N*-palmitoylation of the Hedgehog protein, which is closely related to human diseases caused by abnormal Hedgehog signaling pathways 15. *S*-palmitoylation is also regulated by depalmitoylase due to its dynamic and reversible characteristics. The depalmitoylases found thus far include protein 17A/B/C containing α/β hydrolase domain (ABHD17A/B/C) and ABHD10, palmitoyl-protein thioesterase 1/2 (PPT1/2), and acyl protein thioesterase 1/2 (APT1/2 or LYPLA1/2) 16. Palmitoylase and depalmitoylase jointly maintain the palmitoylation cycle of proteins and are indispensable in regulating protein functions and intracellular signal transduction.

The increasing evidence for functions of palmitoylase and depalmitoylase and continuous identification of palmitoylated substrate proteins have greatly improved our knowledge on the role of palmitoylation in cancer. The palmitoylation cycle directly regulates the functional status of proteins, further changes the signal transduction in tumor cells, and ultimately affects the development of tumors. Moreover, the palmitoylation or depalmitoylation of proteins, including EGFR, RAS, PD-1/PD-L1, etc., are tightly in connection with the progression of tumors and they determine the sensitivity of cancer treatment. This review aims to elucidate the regulatory network of palmitoylation on several signaling pathways closely related to cancer and to provide new ideas and theoretical support for the exploration of tumor targeted therapy.

## 2. Protein palmitoylation regulates EGFR signaling

Abnormal activation of downstream signals of EGFR is linked to cancer occurrence and progression 17. EGFR tyrosine kinase inhibitors (TKIs) have brought hope for clinical targeting of EGFR therapy. However, TKIs resistance has become a clinical hurdle and the mechanism of resistance is not fully understood. In recent years, the discovery of palmitoylation of EGFR receptors has become a new direction for the exploration of TKIs resistance in tumors 18, which may have clinical significance. In tumor cells, the EGFR signaling mainly activates downstream RAS/MAPK, PI3K/ Akt, PLCγ/PKC and JAK/STAT3 signaling pathways, promoting cancer cell growth and metastasis 19. Multiple evidences have shown that palmitoylation is essential in EGFR-mediated downstream signals. Both Lakshmi *et al*. and Azhar *et al.* have revealed that the palmitoylation of EGFR at Cys797 mediated by fatty acid synthase (FASN) promotes its stability and activity in lung cancer 10, 20. The palmitoylation inhibitor (2-bromopalmitate, 2-BP) or FASN inhibitor (orlistat) can inhibit the stability and activity of EGFR, and weaken downstream signal transduction, thereby inhibiting tumor cell growth and increasing the sensitivity of tumor cells to TKIs 10. Eric S *et al.* demonstrated that zDHHC20 also catalyzed the palmitoylation of EGFR at Cys1025 and Cys1122 in breast cancer cells. However, they found that inhibition of zDHHC20-mediated palmitoylation of EGFR contributed to the maintenance of EGFR signaling, promoting the survival, growth and metastasis of tumor cells, and further sensitizing the efficacy of TKIs 18. The above studies demonstrate that palmitoylation has an opposite regulation of EGFR signaling, which complicates the regulation of EGFR signaling by palmitoylation. This may be related to the palmitoylation site of EGFR, tumor types and the mutational environment within the tumor. For example, Kharbanda's group further demonstrated that inhibiting zDHHC20-mediated palmitoylation of EGFR in KRAS mutation-driven lung cancer blocked downstream EGFR signaling and inhibited tumor cell growth, sensitizing the efficacy of PI3K inhibitors 21. Inhibition of EGFR palmitoylation can enhance the efficacy of tumor therapy, showing a better clinical application value. Therefore, it is of great significance to identify EGFR palmitoylase and depalmitoylase and clarify the regulatory mechanism of EGFR palmitoylation in different cancer types and different mutant environments for personalized treatment of cancer patients.

## 3. Protein palmitoylation regulates RAS signaling

The abnormality of downstream signal transduction caused by RAS family gene mutations is associated with the initiation and progression of many cancers 22. The oncogenic activity of RAS requires proper lipid membrane distribution and localization, and palmitoylation has been shown to play an indispensable role in this process 23. The human RAS family contains three RAS genes that encode HRAS, NRAS, KRAS4A and KRAS4B, among which HRAS, NRAS and KRAS4A have been reported to undergo palmitoylation 23. Compared with that of KRAS4A, palmitoylation has a better regulatory effect on membrane localization of HRAS and NRAS 24. The palmitoylated RAS protein is transported from the Golgi body to the cell membrane and it transmits the growth signal from the cell surface growth factor receptor to the intracellular effector protein, causing abnormal cell growth 24. Protein palmitoylation has been found to be necessary in NRAS mutation-driven leukemia 25. The NRAS protein localizes to the plasma membrane and then activates downstream PI3K/Akt, MAPK/ERK and RAL signaling pathways, which is a vital mechanism of leukemogenesis. The abnormal localization of NRAS (Cys186Ser) with the palmitoylation site mutation reduced the potential of NRAS to induce leukemia 25. Among a large number of palmitoyl-transferases, zDHHC9 has been shown to play a crucial role in the palmitoylation of HRAS and NRAS 26. Mutation or down-regulation of zDHHC9 leads to reduced RAS localization to the cell membrane, thereby inhibiting downstream signaling pathway transduction and reducing the oncogenic ability of RAS 27. Further studies showed that phenotypes of NRAS-driven chronic granulocyte mononuclear leukemia and T-cell acute lymphoblastic leukemia were markedly inhibited but not completely attenuated, in zDHHC9-deficient mice 27. Incomplete phenotypic inhibition implies that there may be other palmitoyltransferases *in vivo* such as zDHHC14 with high homology to zDHHC9 that may contribute to NRAS palmitoylation 27. RAS protein palmitoylation is also regulated by depalmitoylase, which releases the depalmitoylated RAS from the cell membrane and returns to the Golgi body for recycling. The RAS depalmitoylase inhibitor Palm B (mainly inhibiting APT1 depalmitoylase activity) increases cell membrane localization of RAS, as well as signal transduction downstream of RAS, enhancing the oncogenic potential of RAS 28. Recent studies have shown that ABHD17A, ABHD17B, and ABHD17C, members of the α/β hydrolase domain 17 family, can depalmitoylate NRAS, and inhibition of expression of the three ABHD17 proteins significantly reduced the depalmitoylation of NRAS 29. These findings suggest that targeted RAS palmitoylation may be a new option for future treatment of HRAS and NRAS-driven tumors. Since there may be more than one palmitoylase or depalmitoylase that regulates post-translation modification of RAS proteins, further identification of other palmitoylases or depalmitoylases is needed for targeting RAS palmitoylation.

## 4. Protein palmitoylation regulates PD-1/PD-L1 signaling

The programmed cell-death protein 1 (PD-1) and the programmed death ligand 1 (PD-L1) are a pair of negative immunosuppression molecules. Together they regulate the balance among T cell activation, immune tolerance and pathological processes 30. PD-1 is mainly located on the surface of macrophages and activated T and B lymphocytes 31. Recently, evidence has demonstrated that PD-1 also appears in liver cancer, melanoma and other tumors, and it promotes the growth of tumor cells by activating mTOR signals 32, 33. PD-L1 locates on the surface of tumor or antigen-presenting cells 34. Highly expressed in some tumor cells, PD-L1 inhibits the function of lymphocytes by binding to PD-1 on the surface of tumor-infiltrating lymphocytes, which is the pivotal mechanism for the immune escape of tumors 35. Thus, by inhibiting the interaction between PD-L1 and PD-1 or decreasing their expression, it may be possible to release a powerful anti-tumor immune response in the body that can effectively treat tumors.

Recent evidences have found that palmitoylation of PD-L1 is necessary for its stability, suggesting that targeting PD-L1 palmitoylation may reduce the level of PD-L1 in tumor cells and improve the efficacy of tumor immunotherapy. Yi *et al.* found that palmitoyltransferase zDHHC9 mediated palmitoylation of PD-L1 on Cys272 in breast cancer, which promoted the stability of PD-L1. Inhibition of zDHHC9-mediated palmitoylation of PD-L1 makes breast cancer cells more sensitive to T cell immune killing 36. Yao *et al.* found that zDHHC3 could also palmitoylate PD-L1 on Cys272 in colon cancer, and the palmitoylated PD-L1 became more stable because it could not be degraded by the ubiquitination endosome pathway, weakening the immune killing effect of T cells in tumors. Their design of a competitive PD-L1 palmitoylation inhibitor, the CPP-S1 peptide, specifically inhibits the palmitoylation of PD-L1, increasing the cytotoxic effect of T cells on tumor cells 37. In addition, FASN has also been reported to promote PD-L1 palmitoylation and expression in cisplatin-resistant bladder cancer cells 38. Because a single protein may be modified by multiple palmitoylases and a single palmitoylase catalyzes the modification of multiple proteins, targeting a single palmitoylase alone may not be optimal. The design of the targeting peptide may provide new inspirations for future targeted palmitoylation therapy. Yao *et al*. also found that PD-1 on the surface of tumor cells could be modified by zDHHC9-mediated palmitoylation, and inhibition of PD-1 palmitoylation significantly reduced the expression of PD-1 on the surface of tumor cells and inhibited tumor growth 39. Therefore, inhibiting the palmitoylation of PD-1 on the surface of tumor cells and immune cells at the same time or combining with PD-1/PD-L1 inhibitors may be an effective treatment strategy. The palmitoylation of PD-L1 and PD-1 highlights the application value of targeted palmitoylation in immunotherapy, further illustrating the regulatory role of palmitoylation in tumor immune microenvironment and identifying palmitoylation of other immune-related proteins is of great significance for tumor immunotherapy.

## 5. Protein palmitoylation regulates Wnt signaling

The Wnt proteins are a class of secreted glycoproteins widely found in cells. It occupies an irreplaceable position in promoting cell proliferation, differentiation and stem cell growth through autocrine or paracrine, and is also bonds closely with the occurrence, development and therapeutic resistance of the Wnt-driven tumors 40. As many as 19 human Wnt proteins are known to mediate two major signaling pathways: canonical Wnt signaling via β-catenin and noncanonical signaling via planar cell polar pathways 41. Studies have shown that most of these Wnt proteins can undergo palmitoylation mediated by Porcn. Inhibition of Porcn-dependent palmitoylation of Wnt proteins can block multiple steps such as Wnt processing, secretion and signal transduction, thereby inhibiting the growth of tumor cells and stem cells 42, 43. Porcn-mediated palmitoylation of Wnt proteins is also regulated by acetyl-CoA carboxylase (ACC) and stearyl CoA desaturase 1 (SCD1). Inhibition of ACC or SCD1 can reduce palmitoylation and signal transduction of Wnt proteins 44, 45. The inhibition of palmitoylation of Wnt proteins shows an optimistic clinical prospect in the treatment of tumors, and researchers have successively developed inhibitors related to palmitoylation of Wnt proteins, including Porcn inhibitors and SCD1 inhibitors. Porcn inhibitors have a potential clinical value in the treatment of a diversity of cancers including head and neck cancer, colorectal cancer, lung cancer, melanoma, breast cancer, and other solid tumors. Currently, four Porcn inhibitors are in Phase I clinical trials, including CGX1321, ETC-159, LGK974, and RXC004 14. SCD1 inhibitors have also shown an ideal inhibition effect on tumor cell growth in lung cancer, breast cancer, prostate cancer and other cancers, but the subsequent toxic and side effects, such as toxicity to sebum cells, limit their clinical applications 4. New SCD1 inhibitors with fewer side effects need to be further developed.

## 6. Protein palmitoylation regulates hedgehog signaling

Protein palmitoylation plays an indispensable role in hedgehog (Hh) signal transduction and the occurrence and development of hedgehog-driven tumors 15. Hedgehog proteins belong to the secretory signaling family. In mammals, Hh signaling is activated by three ligands: Desert Hedgehog (Desert Hedgehog, DHH), Indian Hedgehog (Indian Hedgehog, IHH) or Sonic Hedgehog (Sonic Hedgehog, SHH), of which SHH was found to be most commonly expressed in adult human tissues 46. During embryonic development, these proteins act as morphogenes, forming a signal gradient of long and short distance interactions that mediate embryonic growth and development patterns. Except for a few signals that regulate tumor stem cell-related signaling, Hedgehog protein signaling is usually turned off after embryogenesis 47. However, Hedgehog protein signaling is found to be abnormally activated in medulloblastoma, breast, lung, pancreatic, prostate and other cancers. This abnormally activated Hedgehog signaling has been proved to be linked to Hedgehog-driven tumorigenesis, development and resistance to tumor therapy 48. Multiple studies have confirmed that the Hedgehog acyltransferase (Hhat) -mediated SHH palmitoylation at Cys24 is crucial to the functional and abnormal signaling activation of Hedgehog proteins 9. Targeting Hhat can ablate SHH palmitoylation, thereby inhibiting Hedgehog-driven tumor cell proliferation and stem cell growth. These observations highlight the potential of Hhat as a target for clinical treatment and sensitization of tumors to conventional therapy 15. A high-throughput screening conducted by Petrova *et al*. using a palmitoylation assay based on SHH peptides identified small molecule inhibitors of Hhat, such as RU-SKI 43, that have shown inhibition of SHH palmitoylation *in vitro* 49. In addition, structural analyses of Hhat have key implications for understanding the molecular mechanism of palmitoylation mediated by Hhat and for searching structure-based inhibitors 50, 51. Clinical efficacy testing of Hhat inhibitors and discovery of new inhibitors will contribute to the personalized treatment of tumors in the future.

## 7. Protein palmitoylation regulates Fas/FasL signaling

Fas (CD95/APO-1) is a member of the tumor necrosis factor receptor (TNFR) superfamily. As a transmembrane receptor, it can lead to apoptosis when cross-linked with an agonizing antibody or Fas ligand (FasL) 52. Initially, Fas/FasL was regarded as the death receptor/death ligand system to maintain peripheral immune homeostasis. Later, more evidence has shown that Fas/FasL signaling is in connection with tumor initiation and development 53, 54. Fas/FasL signaling can activate many tumor-related non-apoptotic signals, including MAPK and NF-kB signals, leading to tumor initiation and metastasis 54. If the apoptosis signal activated by Fas/FasL is damaged, tumor cells can develop resistance to apoptosis and eventually lead to tumor progression 55, 56. It has recently been shown that Fas/FasL can undergo a variety of post-translational modifications, in which protein palmitoylation is necessary to regulate Fas/FasL signal transduction 57, 58. Krittalak *et al*. demonstrated that palmitoylation can occur in Fas. This process determines the localization and internalization of Fas, which further induces the assembly of apoptotic signaling complexes and ultimately leads to cell death 59. Christine *et al*. further pointed out that the formation of Fas stable aggregates is the premise for Fas to mediate apoptotic signals through internalization, and the formation of such stable complexes is closely related to Fas palmitoylation, and inhibition of Fas palmitoylation reduces apoptotic signals 60. Guardiola-Serrano *et al*. are the first to demonstrate that FasL can also undergo palmitoylation, which is necessary for the localization of FasL and the mediation of effective Fas/FasL signaling 61. By screening 23 zDHHC family members, Rossin *et al.* found that zDHHC7 was the main palmitoylase of FAS at Cys199. Inhibition of zDHHC7-mediated palmitoylation of Fas can reduce the membrane localization and stability, thereby reducing the sensitivity of colorectal cancer cells to Fas-induced cell death 58. Valeska *et al.*'s studies in primary chronic lymphoblastic leukemia (CLL) showed that acyl protein thioesterases APT1 and APT2 regulated the depalmitoylation of Fas, and APT1 and APT2 in CLL cells caused the depalmitoylation of Fas, leading to Fas-mediated apoptotic resistance 57. These results indicate that palmitoylation of Fas and its ligand FasL plays an indispensable role in Fas/FasL mediated apoptotic signals, which may be a mechanism of apoptotic resistance in tumor cells.

## 8. Protein palmitoylation regulates Scribble (SCRIB) signaling

The SCRIB protein functions as a tumor suppressor that is eventful for regulating cell polarity and Hippo, PI3K/ Akt and MAPK signaling pathways 62, 63. SCRIB cascades Hippo kinase activation, promoting phosphorylation of YES-related proteins (YAP) and transcriptional co-activators (TAZ) with PDZ binding motifs, preventing both from entering the nucleus, thereby inhibiting mitogen-activated protein kinase (MAPK) and Akt signaling activation 63. SCRIB has been reported to be frequently amplified in human cancers and to be incorrectly localized at cell-to-cell junctions, suggesting that its mis-localization may impair its tumor suppressor activity 64. Chen *et al*. confirmed that this localization of SCRIB was regulated by palmitoylation. Abnormal localization of SCRIB mutants (Cys4Ser/ Cys10Ser) with palmitoylation defects leads to destruction of cell polarity. They further established that the correct location of SCRIB on the plasma membrane can activate the Hippo kinase cascade and inhibit MAPK and Akt signaling, and that this plasma membrane localization is regulated by zDHHC7-mediated palmitoylation 65. The palmitoylation of the SCRIB protein is also regulated by the depalmitoylation enzyme APT2, and the APT2 inhibitor ML349 increases the palmitoylation level of SCRIB and promotes its plasma membrane localization 66. However, the effect of protein palmitoylation modification on SCRIB signal on tumor growth and development has not been substantiated *in vivo* or *in vitro*. The potential of targeted SCRIB palmitoylation in the treatment of cancer needs to be further explored.

## 9. Protein palmitoylation regulates DNA damage repair

DNA damage repair is a protective mechanism for maintaining the integrity of the genome, and abnormal DNA damage repair can make the genome unstable, and eventually lead to tumorigenesis 67, 68. Protein palmitoylation is closely associated with the occurrence of melanoma caused by abnormal DNA damage repair. Chen *et al*. found that, after DNA damage occurred in melanocytes, ATR activated the palmitoylation enzyme zDHHC13 through phosphorylation, and then zDHHC13 caused palmitoylation of cysteine on Cys315 of melanocortin-1 receptor (MC1R), a G protein-coupled receptor. It can then drive cAMP-dependent MITF transcription to activate melanin synthesis and enhance the ability of DNA damage repair. However, in certain red-haired individuals with cancer-sensitive mutations such as Arg151Cys and Arg160Trp (red-haired (RHC) mutants), their MC1R Cys315 palmitoylation levels are reduced and cAMP production is reduced, which ultimately leads to a down-regulation of DNA damage repair capacity, thereby increasing the sensitivity of cells to DNA damage 69. MC1R palmitoylation is also regulated by the depalmitoylation enzyme APT2. The APT2 inhibitor ML349 increases MC1R palmitoylation level and signal transduction, promoting DNA damage repair ability, and thereby inhibiting the development of UV-induced melanoma 70. Inhibition of APT2 may help reduce the risk of melanoma, especially in people with red hair who carry cancer-sensitive mutations.

The DNA damage response is also the main regulatory mechanism of cellular sensitivity to DNA damaging drugs and radiotherapy 71, 72. In recent years, a number of protein palmitoyl transferases have been reported to be involved in the DNA damage response in tumor cells, suggesting that protein palmitoylation has enormous potential in regulating DNA damage repair signals. Sudo *et al*. showed that in mesothelioma cells and HEK293 cells, inhibiting zDHHC8 expression resulted in significant genomic instability, defective DNA damage repair, and increased radiosensitivity 73, 74. Cao *et al.* found that primary mouse embryonic fibroblasts (MEFs) treated with 2-bromopalmitate (2BP) showed significant defects in DNA damage repair after ionizing irradiation, and the MEF cells that inhibited zDHHC16 also showed suboptimal DNA damage repair 75. These two studies suggest that zDHHC8 and zDHHC16 may be sensitizing targets for radiotherapy and chemotherapy. However, their palmitoylation substrate proteins and the regulatory mechanism in DNA damage repair remain to be further studied. Recently, Fan *et al.* confirmed that SEDT2, a histone methyltransferase, is a major target of ZDHHC16 in EGFR-mutated glioma cells, and that SEDT2 palmitoylation contributes to the repair of ionizing irradiation-induced DNA damage 76. Gabriele *et al*. also provided evidence for protein palmitoylation to regulate DNA damage repair signals. In *S. cerevisiae*, they found palmitoylation at Cys466 and Cys473 of the PFA4-dependent N-terminal domain of RIF1. By providing a membrane anchor, RIF1 palmitoylation promotes its localization in the internal nuclear membrane, which in turn facilitates non-homologous end-joining (NHEJ) repair 77. Their results not only confirm the key role of RIF1 palmitoylation in DNA damage repair, but also reveal a new mechanism by which cells can promote the selection of DNA damage repair pathways around the nucleus through chelating palmitoylation repair factors on the inner nuclear membrane. At present, the role of protein palmitoylation in DNA damage repair remains to be extensively studied.

## 10. Protein palmitoylation regulates G protein-coupled Receptor (GPCR) signaling

As the largest and most diverse group of membrane receptors (about 900 different members) in the human genome, the GPCR protein family has attracted extensive attention as promising therapeutic targets in many diseases 78. GPCRs can be activated by diverse extracellular molecules such as peptides and transmitters. Subsequently, the activated GPCRs bind to specific sensory proteins, called G proteins, to initiate a series of downstream signals 79. Thus, abnormal GPCR signaling can lead to a variety of disease states, including cancer 80. GPCR signal transduction is mainly affected by the localization or expression of GPCR on the cell membrane, and palmitoylation has been reported to be necessary for plasma membrane localization of GPCRs 81. Early studies have shown that palmitoylation of GPCRs, such as rhodopsin, forms a fourth cytoplasmic loop, which facilitates their localization to the cell membrane, while defects in palmitoylation of GPCRs, such as histamine H2 receptor, CCR5, the thyrotropin receptor, adenosine A1 receptor, Vasopressin V2 receptor, leads to reduced localization to the cell membrane 82. Several GPCRs, such as β2-adrenergic receptor, protease-activated receptor-2(PAR2), D1 dopamine receptor, α2A-adrenergic receptor, Neurotensin receptor-1(NTSR-1), and melanocortin-1 receptor (MC1R) etc., are also modified by palmitoylation 69, 83, 84. The palmitoylation of NTSR-1 promotes its localization to Structured Membrane microdomains (SMDs) and further induces mitogenic signaling in breast cancer cells 83. As mentioned in DNA damage repair (Section 9), palmitoylated-dependent MC1R activation on the cell membrane prevents melanoma. Palmitoylation has also been shown to be essential for the lysosomal degradation of GPCRs on the cell membrane. EGPR internalization, the initiation of lysosomal degradation, has been reported to require palmitoylation regulation. Palmitoylation of PAR2, thromboxane A2 receptor, cannabinoid receptor type 1 and thyrotropin receptor promoted ligand-stimulated internalization, while palmitoylation of vasopressin V1A receptor interfered with ligand-stimulated internalization 84-87. Endosomes formed after internalization, which can recycle EGPRs back to cell membrane or transport them to lysosome for degradation. The lysosomal degradation of PAR1 and CCR5 has been proved to be regulated by palmitoylation, and their palmitoylation-deficient mutants increase their sorting from endosomes to lysosomes and further degradation 88, 89. In addition to palmitoylation of EGPRs, G proteins themselves can also undergo palmitoylation modification, which has an impact on downstream signal transduction of GPCRs. For example, the palmitoylation of Gαq/11 and Gα S, two G protein subunits, are required for membrane anchoring and interaction with GPCRs 90. Palmitoylation of G protein alpha subunit GNA13 regulates its plasma membrane localization and protein stability, thereby activating rho-dependent signaling pathways 91. These results fully demonstrate that palmitoylation plays an important role in the function and signaling of GPCR. However, identification of palmitoylases and depalmitoylases catalyzing GPCRs is largely missing in most studies. Due to the large number of GPCR family members, functional diversity and preferred drug targets, palmitoylation has shown great potential in studies of GPCRs. Thus, further comprehensive exploration of the modification and functional effects of palmitoylation on GPCRs will contribute to the development of novel therapeutic strategies.

## 11. Protein palmitoylation regulates other signaling

In addition to regulating the cellular signal transduction described above, protein palmitoylation has been reported in other signals that are also critical for cancer initiation, development, and treatment. For examples, palmitoylation can regulate estrogen signaling, which is essential for the growth and survival of breast cancer cells 92. Studies have shown that estrogen receptor (ER) α and β, two canonical receptors that regulate estrogen signaling, undergo palmitoylation 93. The palmitoylation of ERα mediated by ZDHHC7 and ZDHHC21 contributes to its stability, plasma membrane localization and proliferation-related signal transduction. Palmitoylation of ERβ results in its localization to the plasma membrane, mediating its anti-proliferation effect 92, 94. In addition, palmitoylation at the C-terminal of GSDME, a member of the cleavage of gasdermin family proteins, can enhance chemotherapeutic drug-induced pyroptosis by modulating GSDME-mediated downstream signal 95. ZDHHC9 facilitates palmitoylation of Glucose transporter (GLUT1) at Cys207 and thus increases its plasma membrane localization, which is critical for glucose supply during glioblastoma (GBM) tumorigenesis 96. Palmitoylation of Smad3 catalyzed by ZDHCH19 promotes the activation of the Transforming Growth Factor-beta (TGF-β) signaling pathway and its interaction with EP300, and enhances expression of mesenchymal markers in the mesenchymal subtype of GBM 97. Elimination of ZDHHC1-mediated palmitoylation of P53 enables tumor cells to evade P53's tumor suppressor signal 98. In recent years, increasingly studies on palmitoylation in cancer have revealed the role of palmitoylation in tumorigenesis. Thus, focusing on the regulatory role of protein palmitoylation modifications in signal transduction will not only lay a foundation for elucidating the mechanism of cancer initiation and development, but also provide a guarantee for better research on anticancer drugs at the molecular level.

## 12. Palmitoylation and tumor therapy

As highlighted, palmitoylation plays an important role in cell signal transduction of tumors, showing a valuable clinical application potential. However, no relevant drugs have been applied in clinical treatment of tumors thus far. It is encouraging that Porcn inhibitors are now in Phase I clinical trials and are expected to bring benefits to cancer patients 14. Other palmitoylase or depalmitoylase inhibitors such as Hhat inhibitors and APT1/2 inhibitors have been developed and applied to basic research of cancer 29, 46. However, the most common DHHC palmitoylases lack specific inhibitors for basic cancer research, making it more difficult to apply to clinical treatment. Although the earliest discovery of 2-Bromopalmitate and the newly discovered N-Cyanomethyln-myracrylamide had an optimal inhibitory effect on DHHC palmitylase 99, they were both inhibitors for a broad spectrum of palmitoylases. There are many challenges in finding inhibitors that block DHHC palmitoylase mediated palmitoylation. Currently, the understanding of how ZDHHC palmitoylase recognizes and catalyzes substrates is very limited, and more studies are needed to clarify the relevant mechanisms. Inhibition of DHHC is complicated by the fact that many members of the DHHC family have overlapping substrates catalyzed by each other or by different subgroups. As a major breakthrough, the three-dimensional structure of human zDHHC20 has recently been reported, providing a new understanding of how the DHHC palmitoylase plays a catalytic role and a new idea for structure-based inhibitor design 100. In addition, as mentioned above, the selective short peptide conjugates designed by Yao *et al.* 37 can specifically bind certain DHHCs and thus inhibit palmitoylation of target proteins, which may be a new direction for the development of DHHC palmitoylation inhibitors in the future.

## 13. Conclusions and future directions

**Figure 3:**
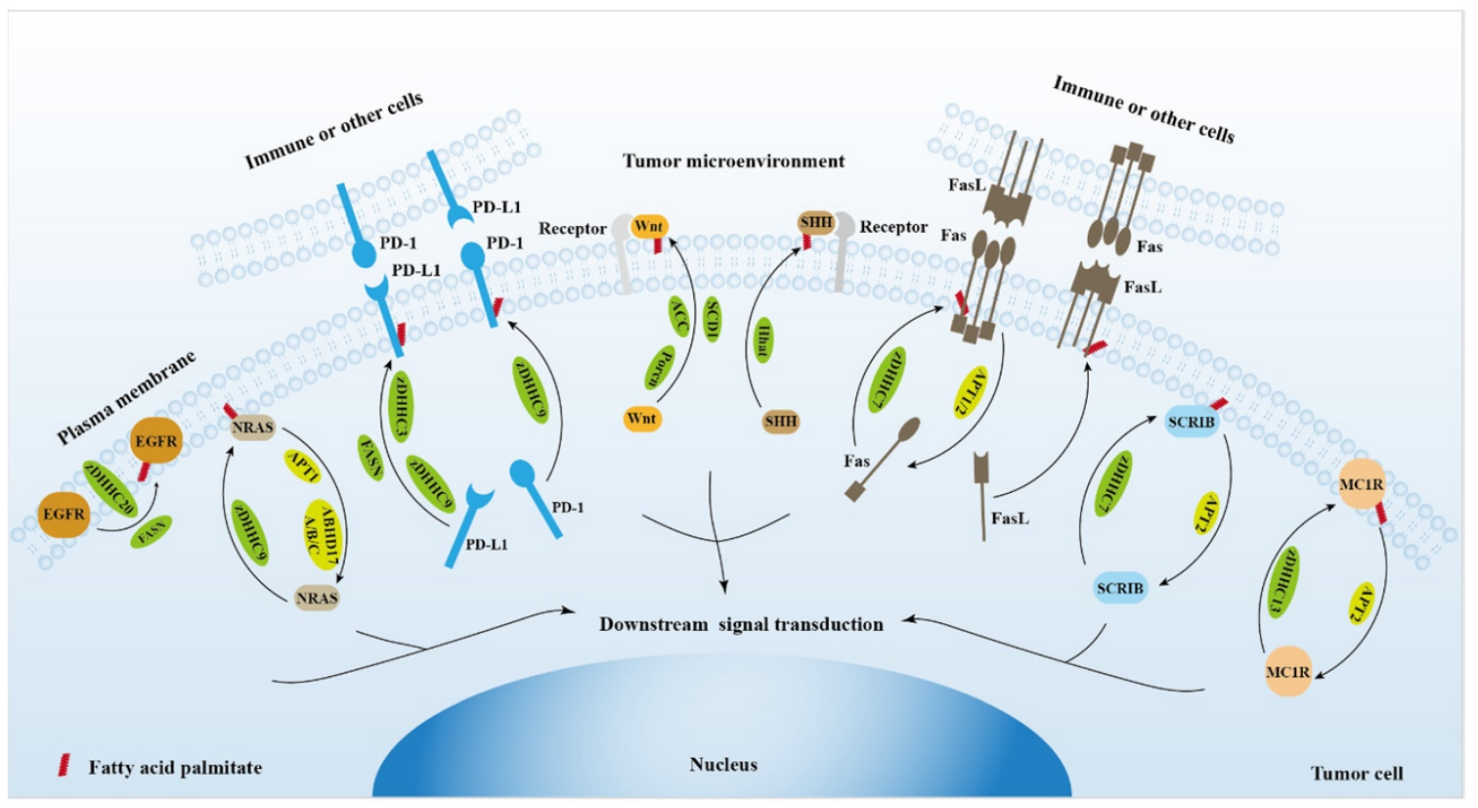
The role of protein palmitoylation in cancer cell signal transduction and tumor microenvironment.

It has been predicted that more than four thousand proteins can be palmitoylated 3, however, the number of proteins identified thus far is still very limited. Thus, continuing to identify palmitoylated proteins, elucidate functional effects of palmitoylation, and identify palmitoylase or depalmitoylase that catalyze these substrates are essential for understanding the critical role of palmitoylation in physiological and pathological settings. At present, most palmitoylated proteins identified are proteins that can bind to the plasma membrane, such as receptors, ligands or secretory proteins. Palmitoylation regulates the localization and function of these proteins on the plasma membrane, allowing tumor cells to receive signals from outside the cell or to transmit signals to the inside the cell 101, 102. These membrane-bound proteins link tumor cells to the microenvironment 103, 104, suggesting that palmitoylation is indispensable for the connection between tumor cells and the tumor microenvironment (Figure 3). Palmitoylation of membrane-bound proteins requires more extensive investigations to discover strategies for tumor prevention and treatment. In addition, some palmitoylases have been also found in the nucleus 37. However, the role of palmitoylated proteins in the nucleus remains to be further investigated. Although palmitoylation has been studied for decades, there is still a lack of clinical drugs for targeted therapy of tumors. The elucidation of the structure of palmitoylase is of great significance for understanding the palmitoylation process and developing structure-based drugs. In addition, due to the fact that palmitoylation of one protein may be catalyzed by multiple palmitoylases, and one palmitoylase can catalyze palmitoylation of multiple substrate proteins, the strategy of blocking palmitoylation of a target protein by inhibiting one palmitoylase may not be feasible, and the design of palmitoylation inhibitors based on the properties of target proteins may provide new ideas for therapeutic strategies targeting palmitoylation.

## References

[1] Conradi C, Shiu A (2018). Dynamics of Posttranslational Modification Systems: Recent Progress and Future Directions. Biophysical journal.

[2] Jiang H, Zhang X, Chen X, Aramsangtienchai P, Tong Z, Lin H (2018). Protein Lipidation: Occurrence, Mechanisms, Biological Functions, and Enabling Technologies. Chemical reviews.

[3] Wang Y, Yang W (2021). Proteome-Scale Analysis of Protein S-Acylation Comes of Age. Journal of proteome research.

[4] Resh MD (2017). Palmitoylation of proteins in cancer. Biochemical Society transactions.

[5] Malgapo MIP, Linder ME (2021). Substrate recruitment by zDHHC protein acyltransferases. Open biology.

[6] De I, Sadhukhan S (2018). Emerging Roles of DHHC-mediated Protein S-palmitoylation in Physiological and Pathophysiological Context. European journal of cell biology.

[7] Won SJ, Martin BR (2018). Temporal Profiling Establishes a Dynamic S-Palmitoylation Cycle. ACS chemical biology.

[8] Gao X, Hannoush RN (2014). Single-cell imaging of Wnt palmitoylation by the acyltransferase porcupine. Nature chemical biology.

[9] Buglino JA, Resh MD (2008). Hhat is a palmitoylacyltransferase with specificity for N-palmitoylation of Sonic Hedgehog. J Biol Chem.

[10] Ali A, Levantini E, Teo JT, Goggi J, Clohessy JG, Wu CS (2018). Fatty acid synthase mediates EGFR palmitoylation in EGFR mutated non-small cell lung cancer. EMBO molecular medicine.

[11] Linder ME, Deschenes RJ (2007). Palmitoylation: policing protein stability and traffic. Nature reviews Molecular cell biology.

[12] Liu Z, Liu C, Xiao M, Han Y, Zhang S, Xu B (2020). Bioinformatics Analysis of the Prognostic and Biological Significance of ZDHHC-Protein Acyltransferases in Kidney Renal Clear Cell Carcinoma. Frontiers in oncology.

[13] Rocks O, Gerauer M, Vartak N, Koch S, Huang ZP, Pechlivanis M (2010). The palmitoylation machinery is a spatially organizing system for peripheral membrane proteins. Cell.

[14] Shah K, Panchal S, Patel B (2021). Porcupine inhibitors: Novel and emerging anti-cancer therapeutics targeting the Wnt signaling pathway. Pharmacological research.

[15] Resh MD (2021). Palmitoylation of Hedgehog proteins by Hedgehog acyltransferase: roles in signalling and disease. Open Biol.

[16] Won SJ, Cheung See Kit M, Martin BR (2018). Protein depalmitoylases. Crit Rev Biochem Mol Biol.

[17] Steuer CE, Ramalingam SS (2015). Targeting EGFR in lung cancer: Lessons learned and future perspectives. Mol Aspects Med.

[18] Runkle KB, Kharbanda A, Stypulkowski E, Cao XJ, Wang W, Garcia BA (2016). Inhibition of DHHC20-Mediated EGFR Palmitoylation Creates a Dependence on EGFR Signaling. Molecular cell.

[19] Bernier J, Bentzen SM, Vermorken JB (2009). Molecular therapy in head and neck oncology. Nat Rev Clin Oncol.

[20] Bollu LR, Katreddy RR, Blessing AM, Pham N, Zheng B, Wu X (2015). Intracellular activation of EGFR by fatty acid synthase dependent palmitoylation. Oncotarget.

[21] Kharbanda A, Walter DM, Gudiel AA, Schek N, Feldser DM, Witze ES (2020). Blocking EGFR palmitoylation suppresses PI3K signaling and mutant KRAS lung tumorigenesis. Science signaling.

[22] Li S, Balmain A, Counter CM (2018). A model for RAS mutation patterns in cancers: finding the sweet spot. Nat Rev Cancer.

[23] Busquets-Hernández C, Triola G (2021). Palmitoylation as a Key Regulator of Ras Localization and Function. Front Mol Biosci.

[24] Lin DTS, Davis NG, Conibear E (2017). Targeting the Ras palmitoylation/depalmitoylation cycle in cancer. Biochem Soc Trans.

[25] Cuiffo B, Ren R (2010). Palmitoylation of oncogenic NRAS is essential for leukemogenesis. Blood.

[26] Swarthout JT, Lobo S, Farh L, Croke MR, Greentree WK, Deschenes RJ (2005). DHHC9 and GCP16 constitute a human protein fatty acyltransferase with specificity for H- and N-Ras. The Journal of biological chemistry.

[27] Liu P, Jiao B, Zhang R, Zhao H, Zhang C, Wu M (2016). Palmitoylacyltransferase Zdhhc9 inactivation mitigates leukemogenic potential of oncogenic Nras. Leukemia.

[28] Dekker FJ, Rocks O, Vartak N, Menninger S, Hedberg C, Balamurugan R (2010). Small-molecule inhibition of APT1 affects Ras localization and signaling. Nat Chem Biol.

[29] Lin DT, Conibear E (2015). ABHD17 proteins are novel protein depalmitoylases that regulate N-Ras palmitate turnover and subcellular localization. eLife.

[30] Salmaninejad A, Valilou SF, Shabgah AG, Aslani S, Alimardani M, Pasdar A (2019). PD-1/PD-L1 pathway: Basic biology and role in cancer immunotherapy. Journal of cellular physiology.

[31] Fourcade J, Sun Z, Benallaoua M, Guillaume P, Luescher IF, Sander C (2010). Upregulation of Tim-3 and PD-1 expression is associated with tumor antigen-specific CD8+ T cell dysfunction in melanoma patients. J Exp Med.

[32] Kleffel S, Posch C, Barthel SR, Mueller H, Schlapbach C, Guenova E (2015). Melanoma Cell-Intrinsic PD-1 Receptor Functions Promote Tumor Growth. Cell.

[33] Li H, Li X, Liu S, Guo L, Zhang B, Zhang J (2017). Programmed cell death-1 (PD-1) checkpoint blockade in combination with a mammalian target of rapamycin inhibitor restrains hepatocellular carcinoma growth induced by hepatoma cell-intrinsic PD-1. Hepatology.

[34] Wang Y, Wang H, Yao H, Li C, Fang JY, Xu J (2018). Regulation of PD-L1: Emerging Routes for Targeting Tumor Immune Evasion. Front Pharmacol.

[35] Kim JM, Chen DS (2016). Immune escape to PD-L1/PD-1 blockade: seven steps to success (or failure). Annals of oncology: official journal of the European Society for Medical Oncology.

[36] Yang Y, Hsu JM, Sun L, Chan LC, Li CW, Hsu JL (2019). Palmitoylation stabilizes PD-L1 to promote breast tumor growth. Cell Res.

[37] Yao H, Lan J, Li C, Shi H, Brosseau JP, Wang H (2019). Inhibiting PD-L1 palmitoylation enhances T-cell immune responses against tumours. Nat Biomed Eng.

[38] Shahid M, Kim M, Jin P, Zhou B, Wang Y, Yang W (2020). S-Palmitoylation as a Functional Regulator of Proteins Associated with Cisplatin Resistance in Bladder Cancer. International journal of biological sciences.

[39] Yao H, Li C, He F, Song T, Xu J (2020). A peptidic inhibitor for PD-1 palmitoylation targets its expression and functions. RSC Chemical Biology.

[40] Routledge D, Scholpp S (2019). Mechanisms of intercellular Wnt transport. Development (Cambridge, England).

[41] Yin P, Wang W, Zhang Z, Bai Y, Gao J, Zhao C (2018). Wnt signaling in human and mouse breast cancer: Focusing on Wnt ligands, receptors and antagonists. Cancer science.

[42] Herr P, Basler K (2012). Porcupine-mediated lipidation is required for Wnt recognition by Wls. Developmental biology.

[43] Hosseini V, Dani C, Geranmayeh MH, Mohammadzadeh F, Nazari Soltan Ahmad S, Darabi M (2019). Wnt lipidation: Roles in trafficking, modulation, and function. Journal of cellular physiology.

[44] Rios-Esteves J, Resh MD (2013). Stearoyl CoA desaturase is required to produce active, lipid-modified Wnt proteins. Cell reports.

[45] Petrova E, Scholz A, Paul J, Sturz A, Haike K, Siegel F (2017). Acetyl-CoA carboxylase inhibitors attenuate WNT and Hedgehog signaling and suppress pancreatic tumor growth. Oncotarget.

[46] Cortes JE, Gutzmer R, Kieran MW, Solomon JA (2019). Hedgehog signaling inhibitors in solid and hematological cancers. Cancer treatment reviews.

[47] Pak E, Segal RA (2016). Hedgehog Signal Transduction: Key Players, Oncogenic Drivers, and Cancer Therapy. Developmental cell.

[48] Pasca di Magliano M, Hebrok M (2003). Hedgehog signalling in cancer formation and maintenance. Nature reviews Cancer.

[49] Petrova E, Rios-Esteves J, Ouerfelli O, Glickman JF, Resh MD (2013). Inhibitors of Hedgehog acyltransferase block Sonic Hedgehog signaling. Nature chemical biology.

[50] Coupland CE, Andrei SA, Ansell TB, Carrique L, Kumar P, Sefer L (2021). Structure, mechanism, and inhibition of Hedgehog acyltransferase. Molecular cell.

[51] Jiang Y, Benz TL, Long SB (2021). Substrate and product complexes reveal mechanisms of Hedgehog acylation by HHAT. Science (New York, NY).

[52] O'Brien DI, Nally K, Kelly RG, O'Connor TM, Shanahan F, O'Connell J (2005). Targeting the Fas/Fas ligand pathway in cancer. Expert opinion on therapeutic targets.

[53] Villa-Morales M, Fernandez-Piqueras J (2012). Targeting the Fas/FasL signaling pathway in cancer therapy. Expert opinion on therapeutic targets.

[54] Peter ME, Hadji A, Murmann AE, Brockway S, Ceppi P (2015). The role of CD95 and CD95 ligand in cancer. Cell Death and Differentiation.

[55] Blomberg J, Ruuth K, Santos D, Lundgren E (2008). Acquired resistance to Fas/CD95 ligation in U937 cells is associated with multiple molecular mechanisms. Anticancer research.

[56] Schattenberg JM, Schuchmann M, Galle PR (2011). Cell death and hepatocarcinogenesis: Dysregulation of apoptosis signaling pathways. Journal of gastroenterology and hepatology.

[57] Berg V, Rusch M, Vartak N, Jüngst C, Schauss A, Waldmann H (2015). miRs-138 and -424 control palmitoylation-dependent CD95-mediated cell death by targeting acyl protein thioesterases 1 and 2 in CLL. Blood.

[58] Rossin A, Durivault J, Chakhtoura-Feghali T, Lounnas N, Gagnoux-Palacios L, Hueber AO (2015). Fas palmitoylation by the palmitoyl acyltransferase DHHC7 regulates Fas stability. Cell death and differentiation.

[59] Chakrabandhu K, Hérincs Z, Huault S, Dost B, Peng L, Conchonaud F (2007). Palmitoylation is required for efficient Fas cell death signaling. Embo j.

[60] Feig C, Tchikov V, Schutze S, Peter ME (2007). Palmitoylation of CD95 facilitates formation of SDS-stable receptor aggregates that initiate apoptosis signaling. The EMBO journal.

[61] Guardiola-Serrano F, Rossin A, Cahuzac N, Lückerath K, Melzer I, Mailfert S (2010). Palmitoylation of human FasL modulates its cell death-inducing function. Cell Death Dis.

[62] Cordenonsi M, Zanconato F, Azzolin L, Forcato M, Rosato A, Frasson C (2011). The Hippo transducer TAZ confers cancer stem cell-related traits on breast cancer cells. Cell.

[63] Johnson R, Halder G (2014). The two faces of Hippo: targeting the Hippo pathway for regenerative medicine and cancer treatment. Nature reviews Drug discovery.

[64] Zhan L, Rosenberg A, Bergami KC, Yu M, Xuan Z, Jaffe AB (2008). Deregulation of scribble promotes mammary tumorigenesis and reveals a role for cell polarity in carcinoma. Cell.

[65] Chen B, Zheng B, DeRan M, Jarugumilli GK, Fu J, Brooks YS (2016). ZDHHC7-mediated S-palmitoylation of Scribble regulates cell polarity. Nature chemical biology.

[66] Hernandez JL, Davda D, Cheung See Kit M, Majmudar JD, Won SJ, Gang M (2017). APT2 Inhibition Restores Scribble Localization and S-Palmitoylation in Snail-Transformed Cells. Cell chemical biology.

[67] Berwick M, Vineis P (2000). Markers of DNA repair and susceptibility to cancer in humans: an epidemiologic review. J Natl Cancer Inst.

[68] Xiao M, Fried JS, Ma J, Su Y, Boohaker RJ, Zeng Q (2021). A disease-relevant mutation of SPOP highlights functional significance of ATM-mediated DNA damage response. Signal transduction and targeted therapy.

[69] Chen S, Zhu B, Yin C, Liu W, Han C, Chen B (2017). Palmitoylation-dependent activation of MC1R prevents melanomagenesis. Nature.

[70] Chen S, Han C, Miao X, Li X, Yin C, Zou J (2019). Targeting MC1R depalmitoylation to prevent melanomagenesis in redheads. Nat Commun.

[71] Stover EH, Konstantinopoulos PA, Matulonis UA, Swisher EM (2016). Biomarkers of Response and Resistance to DNA Repair Targeted Therapies. Clin Cancer Res.

[72] Meng F, Qian M, Peng B, Peng L, Wang X, Zheng K (2020). Synergy between SIRT1 and SIRT6 helps recognize DNA breaks and potentiates the DNA damage response and repair in humans and mice. eLife.

[73] Sudo H, Tsuji AB, Sugyo A, Imai T, Saga T, Harada YN (2007). A loss of function screen identifies nine new radiation susceptibility genes. Biochemical and biophysical research communications.

[74] Sudo H, Tsuji AB, Sugyo A, Ogawa Y, Sagara M, Saga T (2012). ZDHHC8 knockdown enhances radiosensitivity and suppresses tumor growth in a mesothelioma mouse model. Cancer science.

[75] Cao N, Li JK, Rao YQ, Liu H, Wu J, Li B (2016). A potential role for protein palmitoylation and zDHHC16 in DNA damage response. BMC molecular biology.

[76] Fan X, Sun S, Yang H, Ma H, Zhao C, Niu W (2022). SETD2 Palmitoylation Mediated by ZDHHC16 in Epidermal Growth Factor Receptor-Mutated Glioblastoma Promotes Ionizing Radiation-Induced DNA Damage. International journal of radiation oncology, biology, physics.

[77] Fontana GA, Hess D, Reinert JK, Mattarocci S, Falquet B, Klein D (2019). Rif1 S-acylation mediates DNA double-strand break repair at the inner nuclear membrane. Nature communications.

[78] Bar-Shavit R, Maoz M, Kancharla A, Nag JK, Agranovich D, Grisaru-Granovsky S (2016). G Protein-Coupled Receptors in Cancer. International journal of molecular sciences.

[79] Chaudhary PK, Kim S (2021). An Insight into GPCR and G-Proteins as Cancer Drivers. Cells.

[80] Soond SM, Zamyatnin AA Jr (2020). Targeting G protein-coupled receptors in cancer therapy. Advances in cancer research.

[81] Qanbar R, Bouvier M (2003). Role of palmitoylation/depalmitoylation reactions in G-protein-coupled receptor function. Pharmacology & therapeutics.

[82] Patwardhan A, Cheng N, Trejo J (2021). Post-Translational Modifications of G Protein-Coupled Receptors Control Cellular Signaling Dynamics in Space and Time. Pharmacological reviews.

[83] Heakal Y, Woll MP, Fox T, Seaton K, Levenson R, Kester M (2011). Neurotensin receptor-1 inducible palmitoylation is required for efficient receptor-mediated mitogenic-signaling within structured membrane microdomains. Cancer biology & therapy.

[84] Adams MN, Christensen ME, He Y, Waterhouse NJ, Hooper JD (2011). The role of palmitoylation in signalling, cellular trafficking and plasma membrane localization of protease-activated receptor-2. PloS one.

[85] Tanaka K, Nagayama Y, Nishihara E, Namba H, Yamashita S, Niwa M (1998). Palmitoylation of human thyrotropin receptor: slower intracellular trafficking of the palmitoylation-defective mutant. Endocrinology.

[86] Reid HM, Kinsella BT (2007). Palmitoylation of the TPbeta isoform of the human thromboxane A2 receptor. Modulation of G protein: effector coupling and modes of receptor internalization. Cellular signalling.

[87] Oddi S, Stepniewski TM, Totaro A, Selent J, Scipioni L, Dufrusine B (2017). Palmitoylation of cysteine 415 of CB(1) receptor affects ligand-stimulated internalization and selective interaction with membrane cholesterol and caveolin 1. Biochimica et biophysica acta Molecular and cell biology of lipids.

[88] Canto I, Trejo J (2013). Palmitoylation of protease-activated receptor-1 regulates adaptor protein complex-2 and -3 interaction with tyrosine-based motifs and endocytic sorting. The Journal of biological chemistry.

[89] Percherancier Y, Planchenault T, Valenzuela-Fernandez A, Virelizier JL, Arenzana-Seisdedos F, Bachelerie F (2001). Palmitoylation-dependent control of degradation, life span, and membrane expression of the CCR5 receptor. The Journal of biological chemistry.

[90] Wedegaertner PB, Chu DH, Wilson PT, Levis MJ, Bourne HR (1993). Palmitoylation is required for signaling functions and membrane attachment of Gq alpha and Gs alpha. The Journal of biological chemistry.

[91] Xia Z, Zhang X, Liu P, Zhang R, Huang Z, Li D (2021). GNA13 regulates BCL2 expression and the sensitivity of GCB-DLBCL cells to BCL2 inhibitors in a palmitoylation-dependent manner. Cell death & disease.

[92] Le Romancer M, Poulard C, Cohen P, Sentis S, Renoir JM, Corbo L (2011). Cracking the estrogen receptor's posttranslational code in breast tumors. Endocrine reviews.

[93] Anderson AM, Ragan MA (2016). Palmitoylation: a protein S-acylation with implications for breast cancer. NPJ breast cancer.

[94] Pedram A, Razandi M, Deschenes RJ, Levin ER (2012). DHHC-7 and -21 are palmitoylacyltransferases for sex steroid receptors. Molecular biology of the cell.

[95] Hu L, Chen M, Chen X, Zhao C, Fang Z, Wang H (2020). Chemotherapy-induced pyroptosis is mediated by BAK/BAX-caspase-3-GSDME pathway and inhibited by 2-bromopalmitate. Cell death & disease.

[96] Zhang Z, Li X, Yang F, Chen C, Liu P, Ren Y (2021). DHHC9-mediated GLUT1 S-palmitoylation promotes glioblastoma glycolysis and tumorigenesis. Nature communications.

[97] Fan X, Fan J, Yang H, Zhao C, Niu W, Fang Z (2021). Heterogeneity of subsets in glioblastoma mediated by Smad3 palmitoylation. Oncogenesis.

[98] Tang J, Peng W, Feng Y, Le X, Wang K, Xiang Q (2021). Cancer cells escape p53's tumor suppression through ablation of ZDHHC1-mediated p53 palmitoylation. Oncogene.

[99] Lan T, Delalande C, Dickinson BC (2021). Inhibitors of DHHC family proteins. Current opinion in chemical biology.

[100] Rana MS, Kumar P, Lee CJ, Verardi R, Rajashankar KR, Banerjee A (2018). Fatty acyl recognition and transfer by an integral membrane S-acyltransferase. Science (New York, NY).

[101] Leth-Larsen R, Lund RR, Ditzel HJ (2010). Plasma membrane proteomics and its application in clinical cancer biomarker discovery. Molecular & cellular proteomics: MCP.

[102] Erazo-Oliveras A, Fuentes NR, Wright RC, Chapkin RS (2018). Functional link between plasma membrane spatiotemporal dynamics, cancer biology, and dietary membrane-altering agents. Cancer metastasis reviews.

[103] Whiteside TL (2002). Tumor-induced death of immune cells: its mechanisms and consequences. Seminars in cancer biology.

[104] Ma WW, Adjei AA (2009). Novel agents on the horizon for cancer therapy. CA: a cancer journal for clinicians.

